# Supplementary material for: Mutual synchronization of spin-torque oscillators within a ring array
Source: Sci Rep. 2022 Jul 14;12:12030. doi: 10.1038/s41598-022-15483-1 (PMC9283394; doi:10.1038/s41598-022-15483-1)
Supplement: Supplementary file 5 — Supplementary Information 5. [file 41598_2022_15483_MOESM5_ESM.pdf]

Mutual Synchronization of Spin-Torque Oscillators within a Ring Array

M. A. Castro,[1,2] D. Mancilla-Almonacid,[1] B. Dieny,[2] S.Allende,[1] L. D. Buda-Prejbeanu,[2] and U. Ebels[2]
[1] *Universidad de Santiago de Chile (USACH) Departamento de Física, CEDENNA, Avda. Ecuador 3493, Estación Central, Santiago, Chile.*
[2] *Univ. Grenoble Alpes, CEA, CNRS, Grenoble INP, SPINTEC, 38000 Grenoble, France.*

The purpose of this supplementary material is to comment on two points:

I. THE POWER OF OSCILLATIONS

When the STNOs are synchronized, The power of oscillations can be obtained by solving $\sum_{k=1}^{N} \Gamma_{eff}^{k}(p) = 0$ from the Eq. (8), we find:

$$p = -\frac{ajJ_{ave} + \alpha(H_0 - 3H_d)}{4H_d\alpha} - \frac{\sqrt{(a_j J_{ave} + \alpha(H_d + H_0))^2 + 8H_d\alpha^2 M_s \sum_{l=2}^{N} K_3^{1,l}}}{4H_d\alpha} \tag{SP1}$$

where $H_d = M_s(N_z - N_x)$. N_z and N_x are the corresponding demagnetization factors of the circular free layer. In general, the term $H_d + H_0 \gg M_s K_3^{1,l}$, in that way, it is possible to neglect the contribution of K_3 in Eq. (SP1). This assumption only leads to a small shift between the numerical and analytical solution shown in Fig 2.(b). We would like to point out, that different to single STNO equations, the term proportional to p_k^2 cannot be neglected in Eq. (7) , in order to describe the synchronized state correctly.

II. VIDEOS

We included four videos of the in-phase and splay mode for the different cases: two videos for identical current densities ($J_{even} = J_{odd}$) for both modes (cases Fig 1.(e) and 1.(g)), and two videos for non-identical current densities ($J_{even} \neq J_{odd}$) for both modes (cases Fig1.(f) and Fig 1.(h)).

- In-phase.mp4 file: In-phase mode where the subgroups have identical current density, $J_{even} = J_{odd}$.
- Splay.mp4 file: Splay mode where the subgroups have identical current density, $J_{even} = J_{odd}$.
- In-phase_diff.mp4 file: In-phase mode where the subgroups have different current density, $J_{even} \neq J_{odd}$.
- Splay_diff.mp4 file: Splay mode where the subgroups have different current density, $J_{even} \neq J_{odd}$.
